# Electroconvulsive therapy (ECT) during pregnancy: quantifying and assessing the electric field strength inside the foetal brain

**DOI:** 10.1038/s41598-018-22528-x

**Published:** 2018-03-07

**Authors:** Behailu Kibret, Malin Premaratne, Caley Sullivan, Richard H. Thomson, Paul B. Fitzgerald

**Affiliations:** 1 Advanced Computing and Simulation Laboratory (AχL), Department of Electrical and Computer Systems Engineering, Monash University, Clayton, 3800 Victoria, Australia; 2 Monash Alfred Psychiatry Research Centre, Monash University Central Clinical School and the Alfred, 607 St Kilda Rd, Melbourne, 3004 Victoria, Australia; 3 Epworth Healthcare, The Epworth Clinic and Monash Alfred Psychiatry Research Centre, Monash University Central Clinical School and the Alfred, 888 Toorak Rd, Camberwell, 3124 Victoria Australia

## Abstract

Electroconvulsive therapy (ECT) is an effective treatment option for severe mental illness during pregnancy. However, there is little knowledge about the amount of electric field produced inside the foetus, which is important to understand the effects of ECT on the foetal excitable tissues. Thus, in this paper, the electric field strength inside the foetus was computed and compared to the basic restriction of the International Commission for Non-Ionizing Radiation Protection (ICNIRP). A computational human phantom representing a 30-weeks pregnant female, four types of electrode placements and a range of stimulus pulse width (0.25 ms–2 ms) and frequency (10 Hz–140 Hz) were used to compute the electric field inside the foetus. A linear relationship between the maximum electric field inside the foetal brain and the electrode current was derived. The results suggest that, considering the maximum current output, pulse width, and frequency range of constant-current ECT devices, the electric field produced inside the foetal brain is most likely below the ICNIRP basic restriction. This is based on the practical scenario of a 30-weeks foetus with a bottom-up and head-down foetal position and the mother taller than 1.62 m.

## Introduction

Electroconvulsive therapy (ECT) is a psychiatric treatment in which electric currents are applied through scalp electrodes to induce seizures in anesthetized patients. ECT is often used as a last line of intervention for major depressive disorder, mania, and catatonia^1^. It is also an effective treatment option for several psychiatric disorders in pregnant patients^2^. An extensive review of the reported cases of ECT performed on pregnant patients shows, out of the 339 collected reports, 11 cases represented foetal complications likely related to ECT^3^. The reported foetal complications include transient foetal arrhythmia, a foetal death secondary to status epilepticus, and a miscarriage. In another review, nearly one third of the cases reported adverse events, such as, foetal heart rate reduction, uterine contraction, and premature labor, from the total cases of 169 pregnant patients who received ECT^4^.

Despite the increasing interest in understanding the adverse effects of ECT during pregnancy, there is little knowledge about the amount of electric field produced inside the foetus. The knowledge of the electric field inside the foetus is crucial to understand the effect of ECT on the foetal excitable tissues, such as, the muscular tissue or tissues in the central and peripheral nervous system. Within this context, for the first time, we numerically computed the electric field inside the foetal tissues by utilizing the computational model of a 30-weeks pregnant female. The computed electric field was also compared to the basic restriction of the International Commission for Non-Ionizing Radiation Protection (ICNIRP)^5^, which are limits defined to protect from the effects of low frequency electromagnetic fields, such as, stimulation of excitable tissues.

In this paper, the electric field from ECT was numerically computed inside the computational model of a 30-weeks pregnant female that was developed by merging the mesh model of a non-pregnant female and a 30 weeks old foetus. The computed electric field was related to the amplitude of rectangular biphasic stimulation current waveforms, which were used to compare the electric field inside the foetal brain to the ICNIRP basic restriction.

## Materials and Methods

### The pregnant female computational model

**Figure 1:**
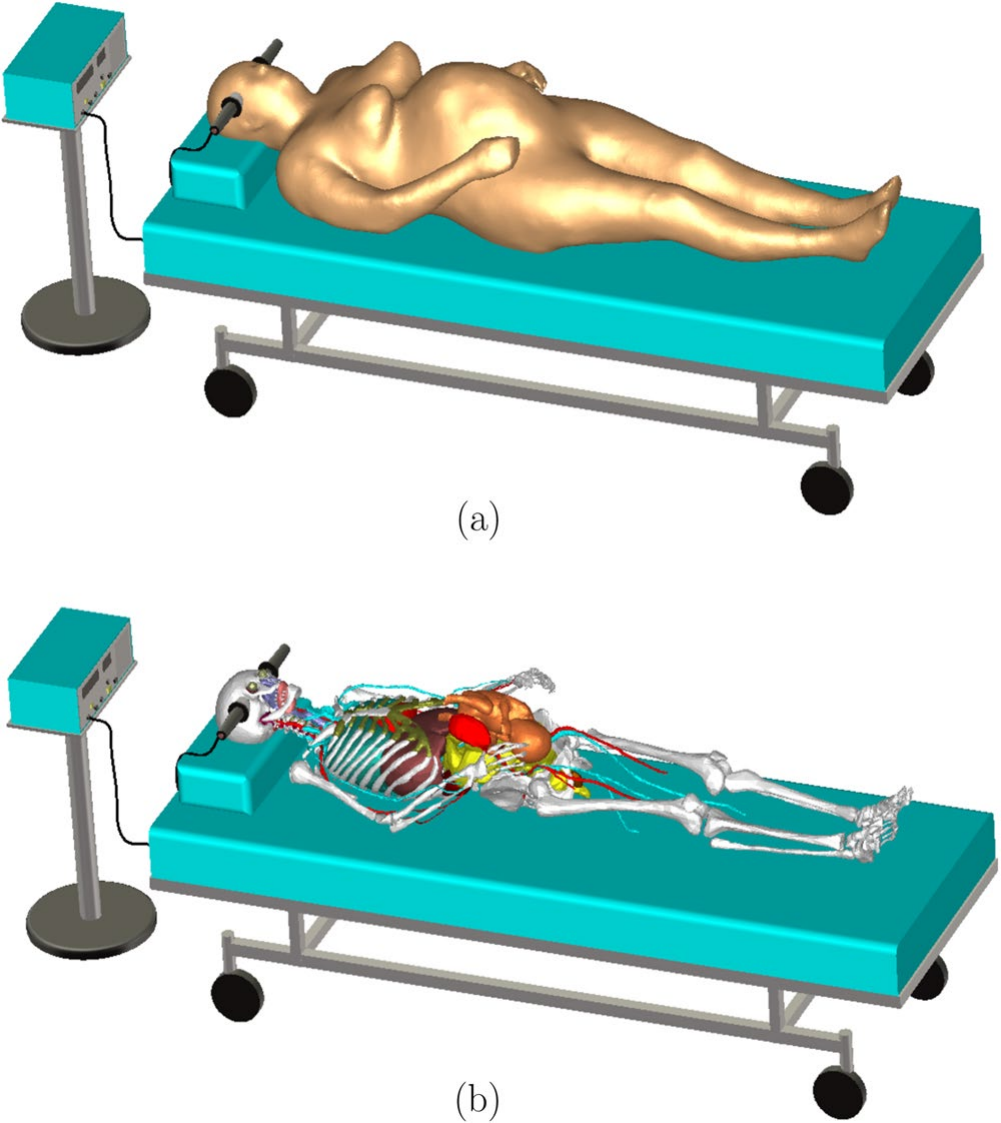
The pregnant female phantom in typical ECT setting. In (**b**), skin, fat, muscle, uterus, and amniotic fluid were hidden for better view of the inner organs and the foetus.

**Figure 2:**
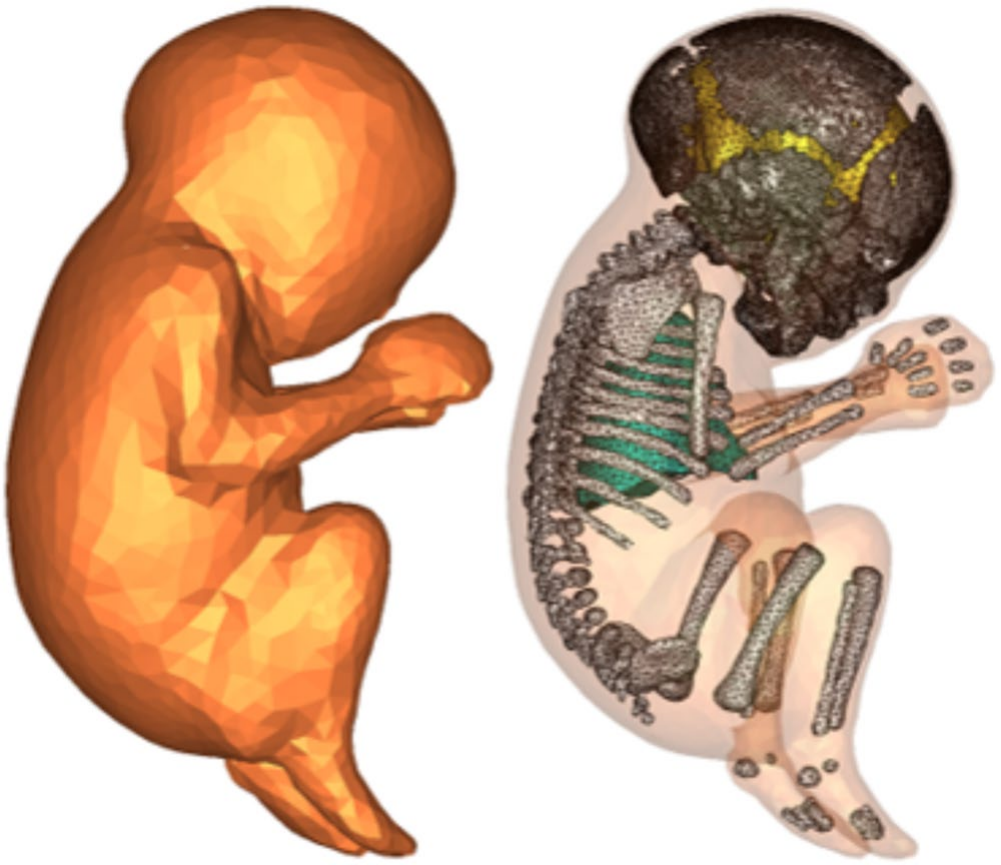
The foetus phantom.

The problem of computing the electric field distribution inside the foetus requires a full-body pregnant computational model since the ECT current is applied on the mother’s scalp. However, the whole-body medical image datasets of a pregnant patient, which are needed to develop a full-body computational model, are not available since it is unethical to unnecessarily expose the foetus to imaging radiation. Consequently, most of the pregnant female computational models available in the world are hybrid models that were built by combining a full-body non-pregnant female model with the model of foetus or utero-foetal unit that was built from obstetric images^6–14^. Moreover, the development of most of such models required the manual translation and deformation of the maternal organs to place the utero-foetal unit. Similarly, we built the pregnant female computational model by merging the mesh model of a non-pregnant female (obtained from http://www.nevaelectromagnetics.com) and a foetus (obtained from http://femonum.telecom-paristech.fr/). The non-pregnant female mesh model (VHP Base 3.0), which has 26 individual tissues, was constructed from a cryosection image dataset of a female cadaver of height 162 cm. The VHP Base 3.0 was modified by stretching the skin, fat and muscle tissues at the abdomen by referring to the anatomical atlas of a pregnant female^15^. Also, other body parts, such as the intestine, liver, stomach, and blood vessels were deformed to place the uterus. The triangular surface meshes were made not to have non-manifold faces, non-manifold vertices and self-intersections. The development of the utero-foetal mesh model is discussed in^7,16^. The model consists of brain, lungs, skeleton, soft tissues, uterus and uterus content. The uterus content consists of uterus wall, placenta and amniotic fluid. The placenta was modeled with location and shape similar to that of the anatomical atlas used. Figures 1 and 2 show the pregnant female computational model in a typical ECT setting and the foetus model, respectively.

**Figure 3:**
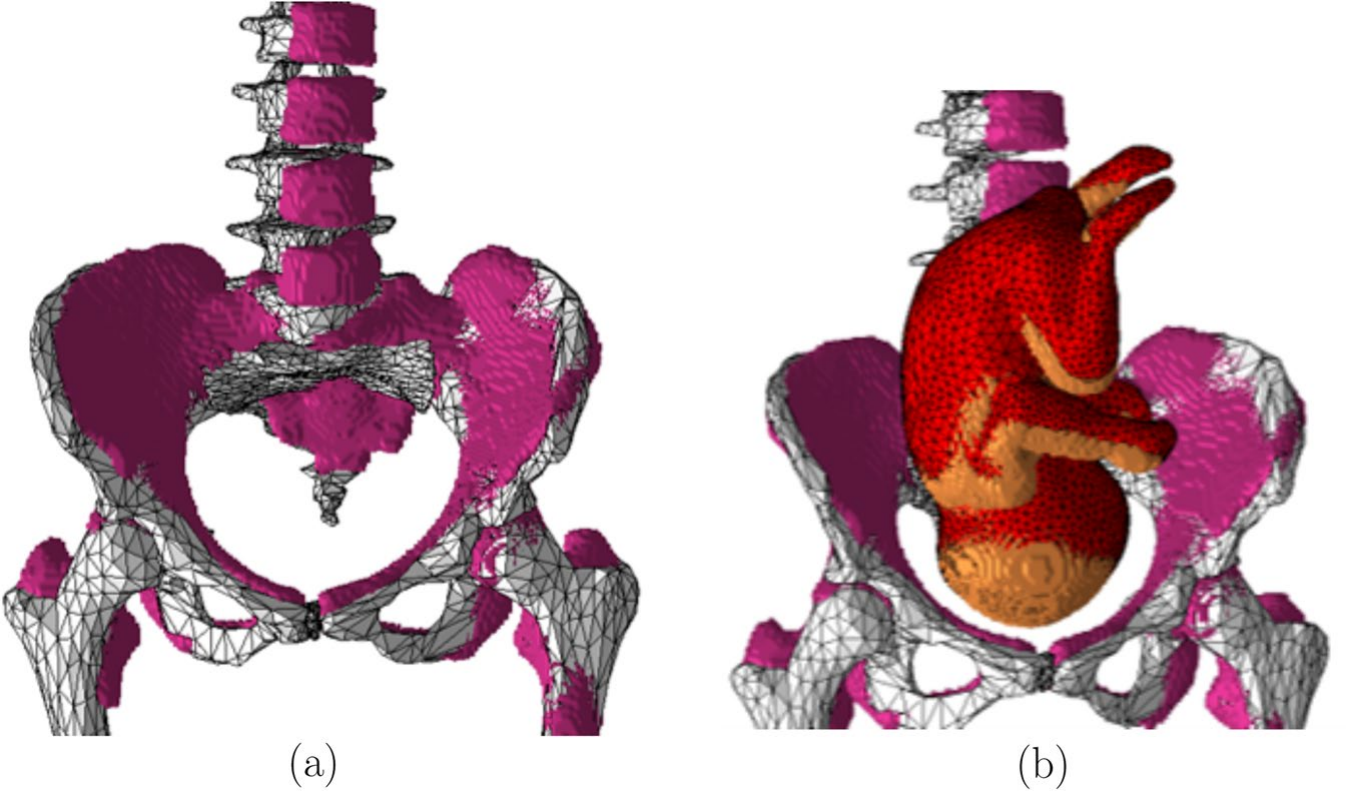
(**a**) The voxel based pelvis bone of the FEMONUM model (purple) overlying on the mesh based pelvis bone of our model (grey). (**b**) The voxel based foetus (28 weeks) of the FEMONUM model (orange) overlying on the mesh based foetus of our phantom (red).

**Figure 4:**
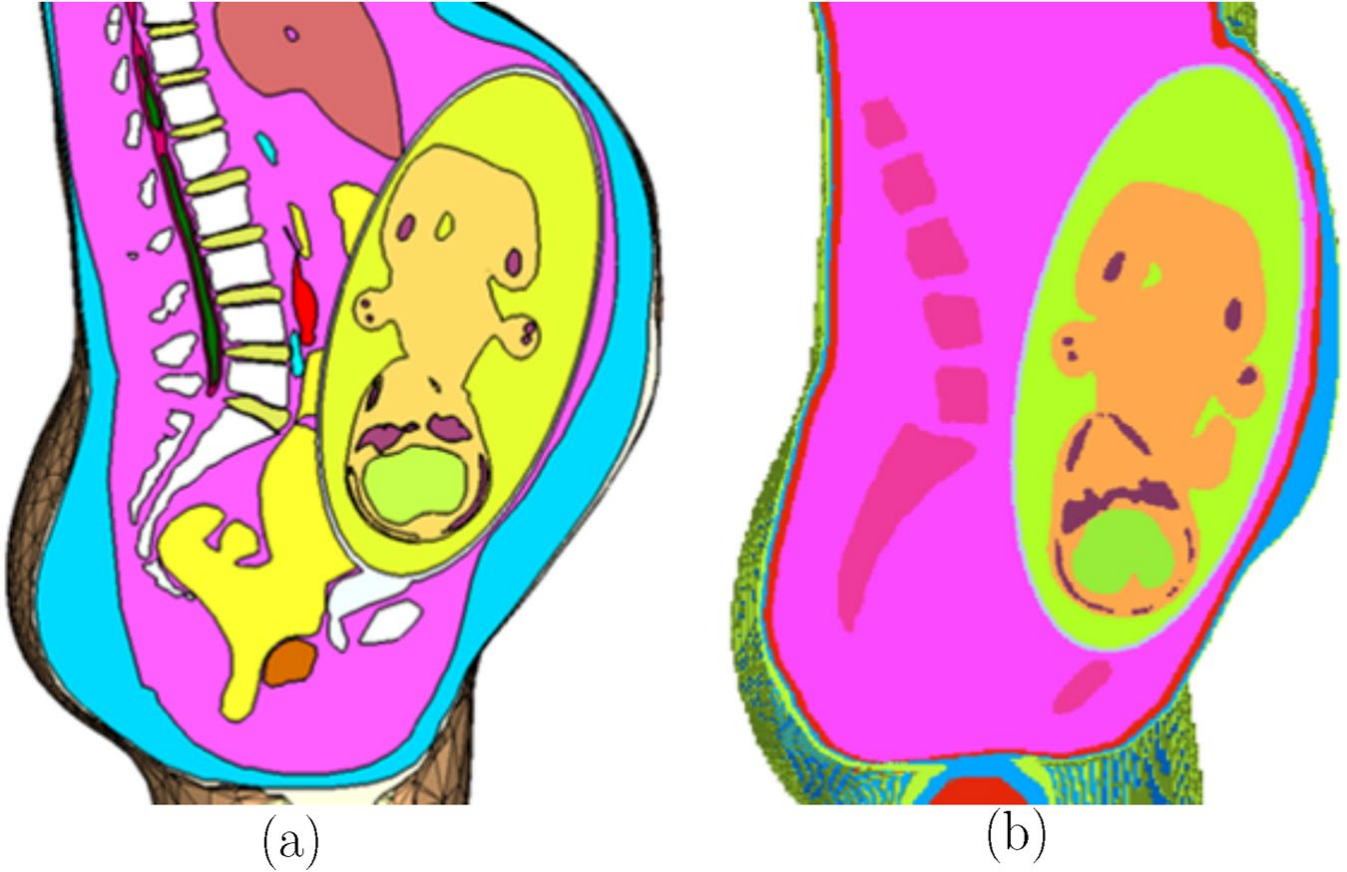
Comparison of our model (**a**) to the 32 weeks FEMONUM model (**b**) in sagittal section.

The computational model was validated by comparing it to the pregnant computational models developed by the FEMONUM project (http://femonum.telecom-paristech.fr/projects.html), which were made available to the scientific community after passing an evaluation by an obstetrician and a group of paediatric radiologists^7,16^. The models obtained from the FEMONUM project are voxel based that consist of homogeneous pregnant woman envelopes (generated from a 3D model provided by DAZ 3D http://www.daz3d.com), pelvis bone with some vertebrae, and utero-foetal units representing 28 and 32 weeks of gestation. The pelvis bone was used as a visual landmark to correctly place the utero-foetal unit inside the woman envelope. Therefore, we also used the voxel pelvis bone as a landmark by overlying it on the mesh based pelvis bone of our model to compare the positions of the utero-foetal units. Figure 3(a) shows the voxel pelvis bone overlying on the mesh based pelvis bone. It is seen that the two pelvis bones match fairly accurate despite being developed from the images of different females. Since a 30 weeks pregnant voxel model was not provided by the FEMONUM project, we used the 28 weeks voxel model for comparison as shown in Fig. 3(b). As expected, the 28 weeks foetus is slightly smaller than the 30 weeks model we used; but, their position and orientation match with reasonable accuracy. Figure 4 shows a comparison of our model to the 32 weeks FEMONUM model. It is seen that the locations of the utero-foetal units with respect to the available maternal organs also agree well. The large fat tissue (blue) in the abdominal area of our model is in proportion to the fat volume in other areas of the body. The original non-pregnant model (the VHP Base 3.0) is categorized as obese; therefore, the fat volume on the abdominal area was preserved when it was deformed to fit the utero-foetal unit.

### The computational technique

In the spectral content of ECT pulses, the tissue conduction current density is much larger than the displacement current density; thus, the electric potential distribution *ϕ* was computed by neglecting the capacitive effects and solving the continuity equation

1 $$\nabla \cdot \sigma (-\nabla \varphi )=0$$

where *σ* is the conductivity of tissues. Looking at the typical ECT setting in Fig. 1, the effect of the mattress or cloth can be ignored since it is an electric insulator (*σ* = 0). Also, ECT electrodes are isolated from the mains ground; thus, the capacitive effect of the bed frame can also be neglected. Therefore, the boundary of the problem was limited to the skin surface. The expression in (1) was solved by imposing a Dirichlet boundary condition on the skin surface underlying the electrodes and a Neumann boundary condition on the rest of the skin surface. The computational domain was discretized into tetrahedral volumetric meshes that are suitable to solve the expression in (1) using finite element analysis. It was assumed that the electrode current has a rectangular biphasic waveform so that its amplitude was used to compute the potential *ϕ*. The computed potential was used to calculate the electric field **E** = −∇*ϕ* that has similar waveform to the electrode current. The stationary current solver of CST Studio (CST, Darmstadt, Germany) was used to solve (1), which allows the use of the electrode current amplitude (electrode current density) as the initial condition.

The conductivity of the maternal tissues was obtained from the online IT’IS database (https://www.itis.ethz.ch/virtual-population/tissue-properties/downloads/database-v3-1/). The anisotropic conductivities of muscle, cortical bone, cerebellum, grey matter, and white matter provided in the database were also used. However, the data for the dielectric properties of foetal tissues is rare. Therefore, foetal tissue conductivity values obtained from the relationship between tissue water content and aging was used. It is known that tissue water content decreases with age, which also causes a general decrease in the conductivity of tissues^17^. Dimbylow^6^ proposed a formula that relates the ratio of foetal conductivity, *σ*_*F*_, to the adult, *σ*_*A*_ based on the volume fraction of water in foetal, *p*_*F*_, and adult, *p*_*A*_, body tissues as

2 $${\sigma }_{F}/{\sigma }_{A}={p}_{F}(3-{p}_{A})/({p}_{A}(3-{p}_{F})).$$

The water fraction in the foetal body tissues was taken from the reference values in the ICRP 89^18^ (*p*_*F*_ = 0.807 for a 30 weeks foetus) and the total body water for the reference adult female (*p*_*A*_ = 0.5) from ICRP report 23^19^. Accordingly, for a 30 weeks foetus, the conductivity of the foetal tissue is 1.84 times more than that of the adult female. Since the foetal brain in our model was considered as a single tissue and was not segmented to different parts, its conductivity was related to the average of the conductivities of the adult’s isotropic grey matter and white matter. Moreover, since the foetal lungs are not filled with air, their conductivity was related to the adult’s deflated lungs. Also, the conductivity of the other tissues was related to the conductivity of similar tissues of the adult, such as, the foetal skeleton to the adult’s cortical bone, and the foetal soft tissues to the adult’s muscle. The conductivity of the placenta was assumed to be equal to the conductivity of blood due to the similarity of their composition^20^. The conductivity of the amniotic fluid was taken as 1.27 S/m^21^.

An accurate representation of the skin impedance is crucial for the computation of the dynamic impedance encountered during ECT. It is well known that the dynamic impedance decreases when the stimulation voltage of ECT increases. In other types of electrical transcutaneous stimulations, similar nonlinear impedance variations were attributed to the changes in the characteristics of the stratum corneum underneath the electrode interface^22^. One of the causes of such changes is the mechanism of electroporation of the skin, which is the phenomenon where the membrane permeability to ions and macromolecules is increased when exposed to a high electric field^23^. In other words, the skin conductivity changes with the applied electric field or the stimulation voltage. Experimental data suggest that the conductivity is linearly related to the current density^24^. Thus, the conductivity of the skin in contact with the electrode surface *σ*_*es*_ can be expressed as

3 $${\sigma }_{es}={\sigma }_{e}+{\sigma }_{s}=k{J}_{es}+{\sigma }_{s}$$

where *σ*_*e*_ = *kJ*_*es*_ is the increased conductivity due to the current density *J*_*es*_; *k* is a proportionality constant; and *σ*_*s*_ is the conductivity when the applied current is very small. In other words, *σ*_*e*_ is responsible for the reduced dynamic impedance and *σ*_*s*_ is responsible for the large static impedance. The conductivity relation (3) was applied to the expression in (1) and the resulting nonlinear equation was solved using the stationary current solver of CST Studio^®^.

**Figure 5:**
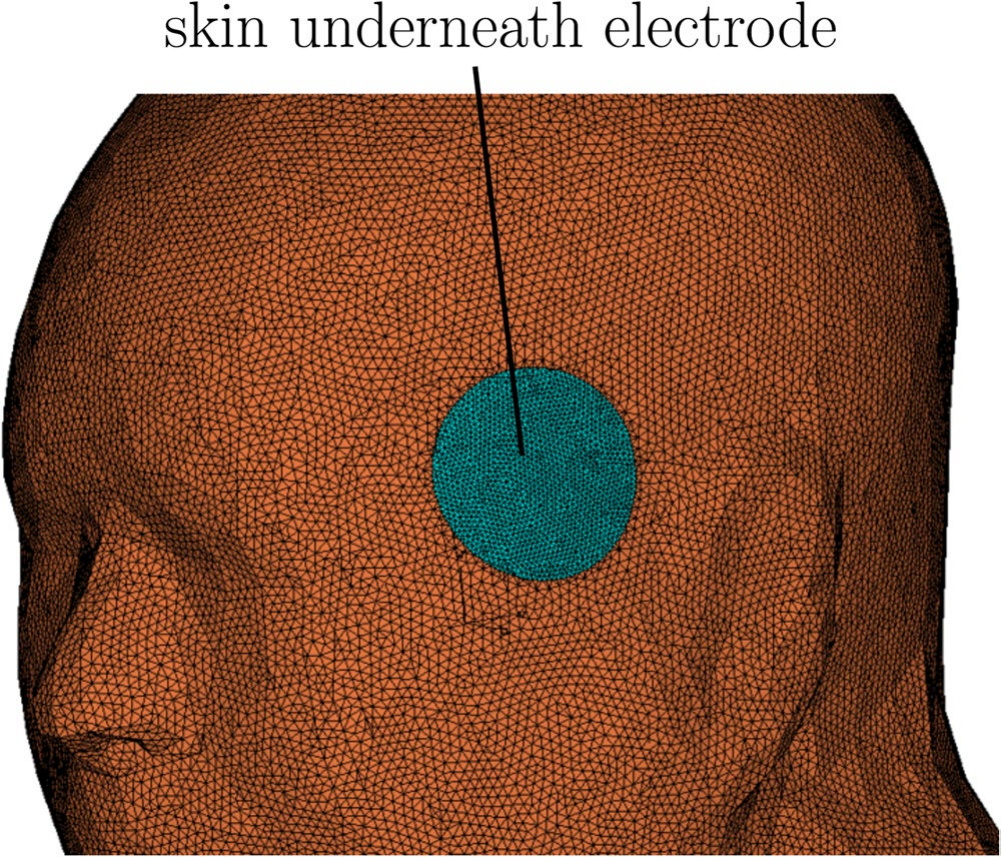
The skin underneath the electrodes (the blue) were separated from the rest of the skin (brown) and assigned a current density dependent conductivity.

Assuming the current density *J*_*es*_ is uniform on an area *A* of the skin underlying the electrodes that has a thickness of *d*, the expression in (3) can be written in terms of the skin conductance *G*_*s*_ and the current *I* as

4 $${G}_{s}=(kI+A{\sigma }_{s})/d=aI+b$$

where *a* and *b* are constants that are used to calculate *k* and *σ*_*s*_. Luna *et al*.^25^ discussed the technique of estimating the subject specific coefficients *a* and *b* from transcutaneous stimulation measurement data. We used *a* = 25.9 mS/A and *b* = 37.8 *μ*S, which was estimated for a single subject in their study^25^, to calculate the conductivity of the skin in contact with the electrodes. In the computational model, the skin underlying the stimulus electrodes was separated, meshed at higher resolution, and assigned a current density dependent conductivity as shown in Fig. 5.

**Figure 6:**
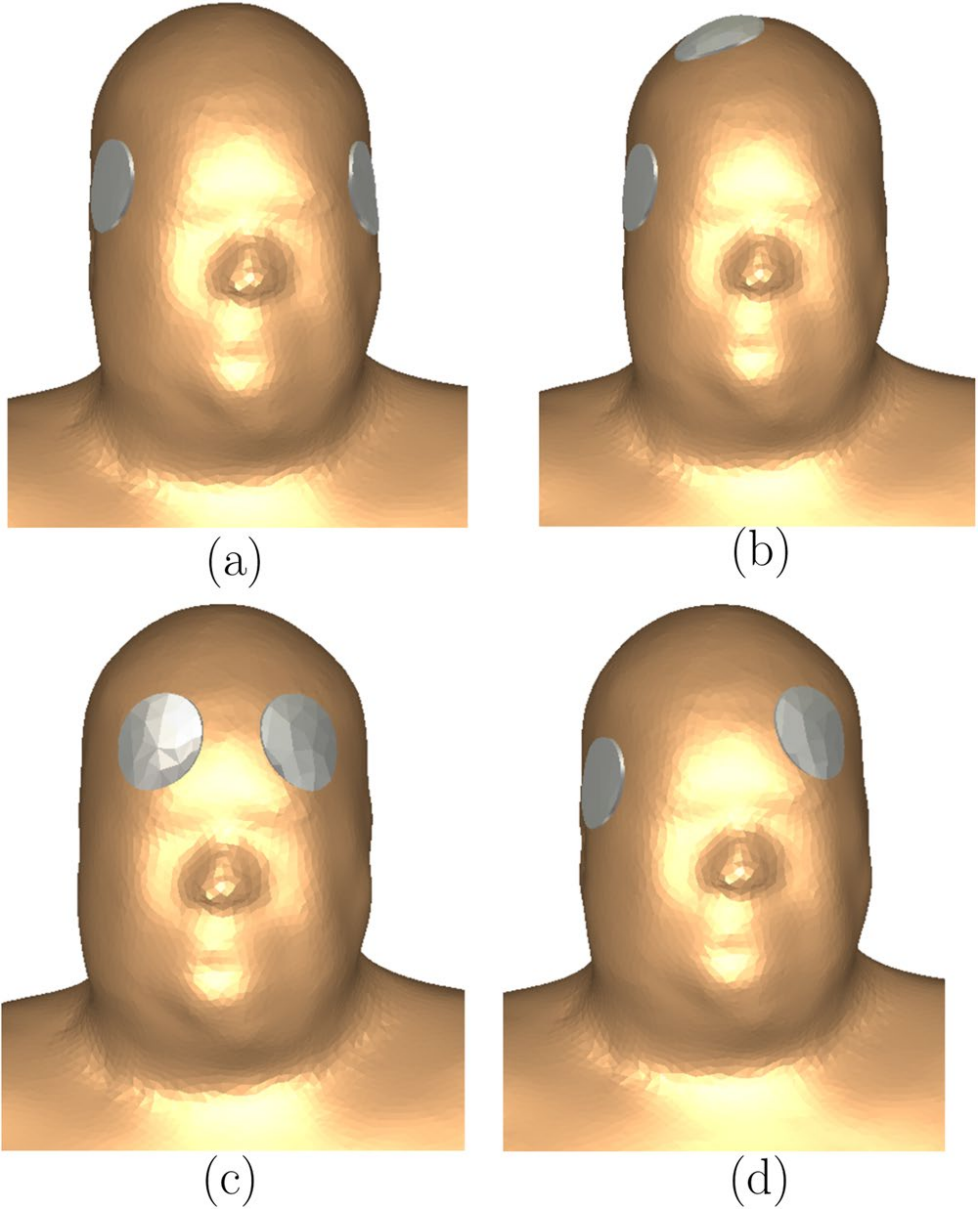
The electrode placement considered (**a**) bilateral (BL), (**b**) right unilateral (RUL), (**c**) bifrontal (BF), and (**d**) left anterior right temporal (LART).

We simulated the four electrode placements shown in Fig. 6, which are, the bilateral (BL), right unilateral (RUL), bifrontal (BF), and the left anterior right temporal (LART). The standard circular electrodes of diameter 5 cm (2 inches) were used and the surface of the underlying skin was assumed to be equipotential.

## Results

**Figure 7:**
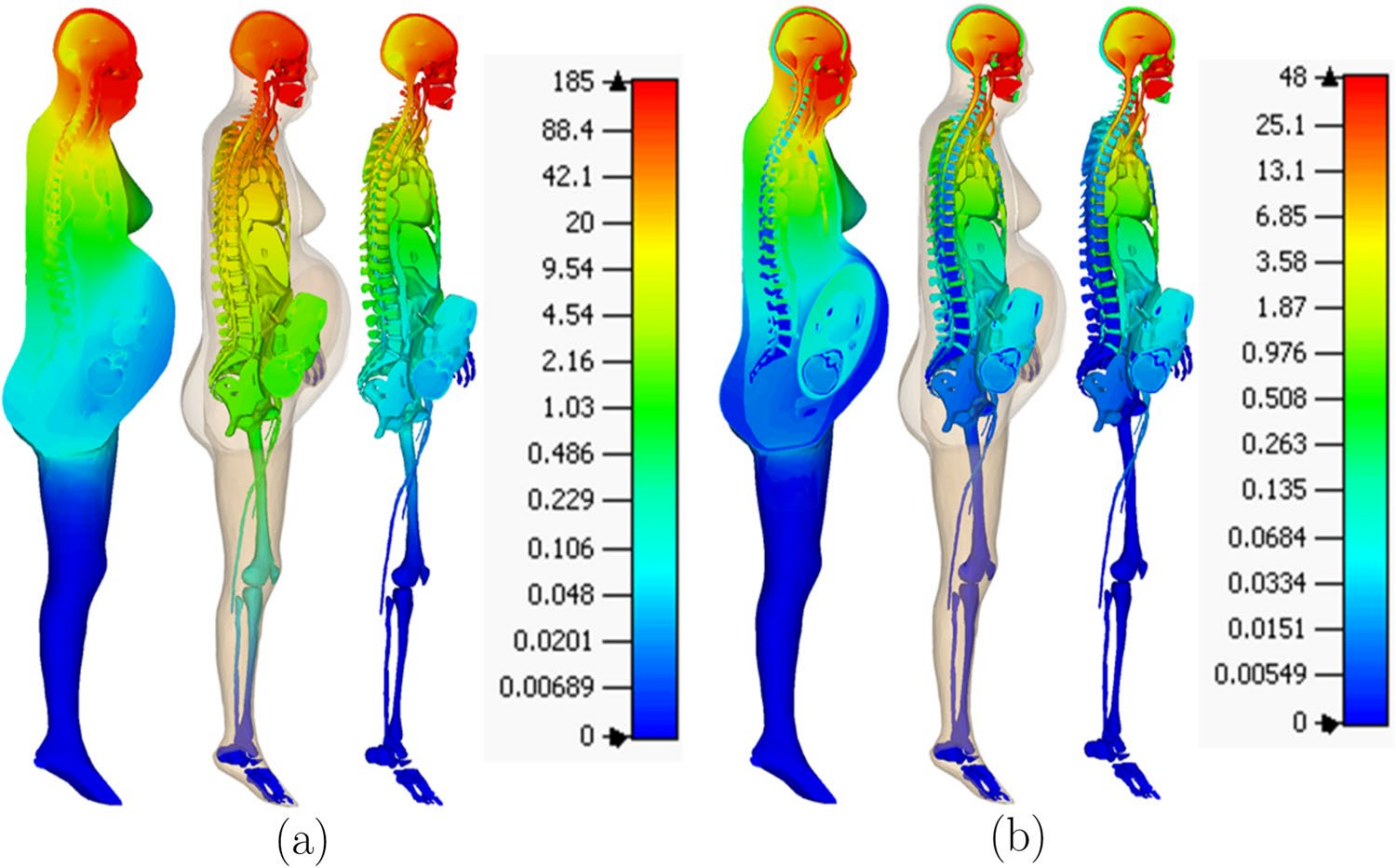
The electric field (**a**) and corresponding current density (**b**) on the sagittal section for bilateral ECT when 0.9 A current is applied on the electrodes.

**Figure 8:**
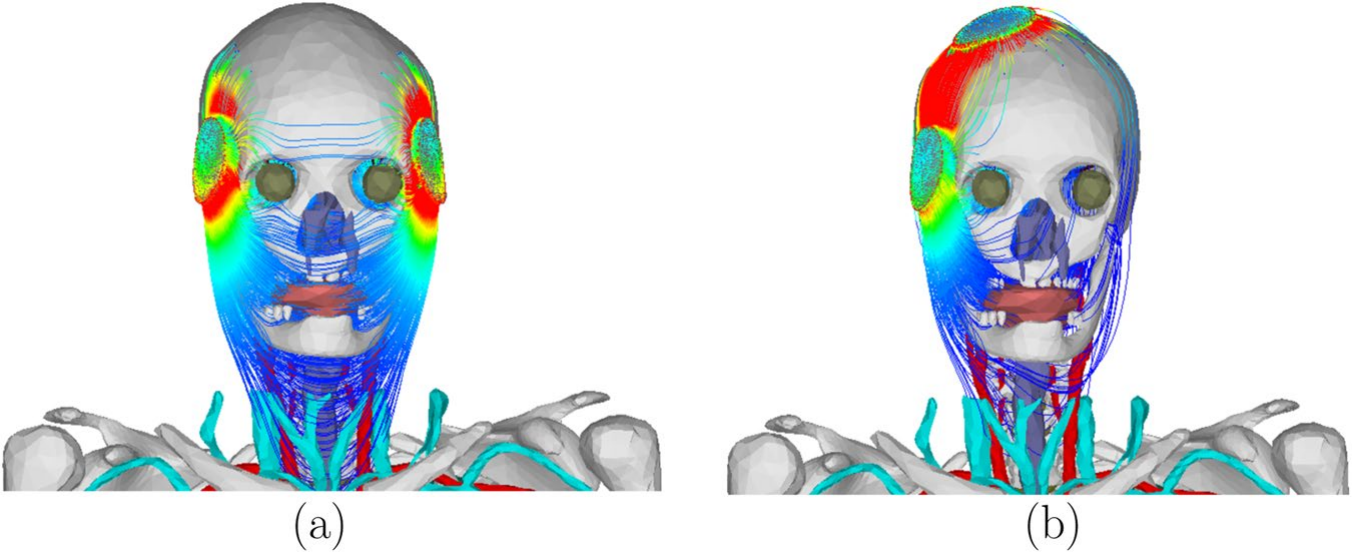
The current density lines for (**a**) bilateral and (**b**) right unilateral electrode placement. The current from the bilateral electrodes is distributed farther down the soft tissues in the neck.

In order to represent the maximum current output of constant-current ECT devices, electrode current of 0.9 A was used to compute the electric field and the corresponding current density inside the tissue for each electrode placement. Figure 7 shows the electric field and the current density for the bilateral electrode placement. Table 1 shows the dynamic impedance and the maximum electric field on the foetal brain for each electrode placement. The difference in the impedance is attributed to the difference in the current path for each electrode placement. For example, the small impedance for BL electrode placement is due to the small impedance of the soft tissues on the current path as shown in Fig. 8(a). On the other hand, the large impedance for RUL electrode placement is due to the large impedance of the skull and the small cross-sectional area of the scalp (skin and subcutaneous fat) that are on the current path as shown in Fig. 8(b). From the expression of the conductance in (4), the impedance of the skin underlying the electrodes was 42.83 Ω. The impedance of the other tissues (including the skin that is not underneath the electrodes) was 201.72 Ω for BF, 171.61 Ω for BL, 201.61 Ω for LART, and 243.84 Ω for RUL. The percentage impedance contribution of the other tissues was 82% for BF, 80% for BL, 82% for LART, and 85% for RUL.

**Table 1:** The voltage, dynamic impedance, and the maximum electric field in the foetal brain (*E*_*a*_) for the four electrode placements.

electrode	voltage (V)	dynamic impedance (Ω)	*E*_*a*_ (V/m)
BF	220	244.55	0.0137
BL	193	214.44	0.0495
LART	220	244.44	0.0320
RUL	258	286.67	0.0204

**Figure 9:**
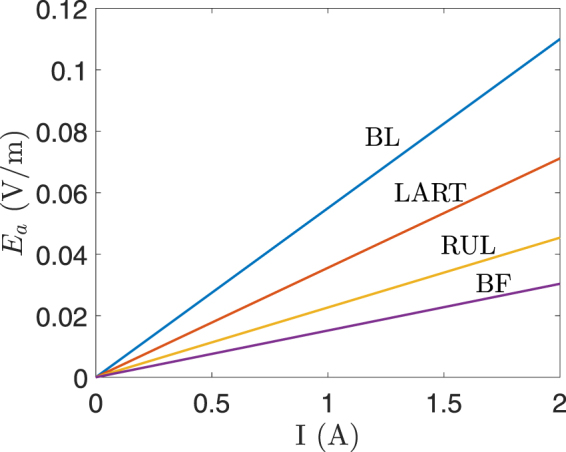
The maximum electric field in the foetal brain (*E*_*a*_) versus the electrode current *I* for the four electrode placements.

The conductivity of the skin underlying the electrodes was calculated based on the values of *a* and *b* (see (4)) that were estimated for a single subject^25^. The contribution of the static component of the conductivity (*b*) is negligible at the electrode current amplitude used in ECT, such as, 0.9 A. The values of *a* and *b* depend on the subjects and they determine the accuracy of the computed dynamic impedance and the voltage drop across the skin. In other words, the values of *a* and *b* are important when an electrode voltage is used as the initial condition in (1) since the dynamic impedance determines the electrode current. However, when an electrode current is used as the initial condition, the computed electric field in the other tissues (excluding the skin underlying the electrodes) is independent of the value of *a* and *b*. This implies that for constant-current ECT devices, the electric field in the other tissues remains unchanged irrespective of the fluctuation in the dynamic impedance of the underlying skin. Therefore, a simple linear relation was derived for the maximum electric field in the foetal brain *E*_*a*_ (V/m) and the electrode current *I* (A) as *E*_*a*_ = *γI*, where *γ* is the constant of proportionality and its value is 0.055 for BL, 0.0152 for BF, 0.0227 for RUL, and 0.0356 for LART. Figure 9 shows the plot of *E*_*a*_ versus the electrode current *I* for the four electrode placements.

## Discussion

The maximum electric field in the foetal brain was compared to the basic restriction set by the ICNIRP. The basic restrictions of ICNIRP in the frequency range of 1 Hz–100 kHz were defined based on the well-known effects of the low frequency electric and magnetic fields, which are, the stimulation of electrically excitable nerve and muscle tissues, the perception of surface charges, and the induction of retinal phosphenes^5^. The ICNIRP guideline states that the evidences for the neurobehavioural, developmental or reproductive effects due to low frequency electromagnetic field exposure are much less clear or very weak. ICNIRP recommends basic restrictions of the electric field in the brain not exceeding 0.1/f V/m for 1 Hz–10 Hz, 0.01 V/m for 10 Hz–25 Hz, 4 × 10^−4^*f* V/m for 25 Hz–1000 Hz, and 1.35 × 10^−4^*f* V/m for 3 kHz–10 MHz, where *f* is electric field frequency in Hz.

**Figure 10:**
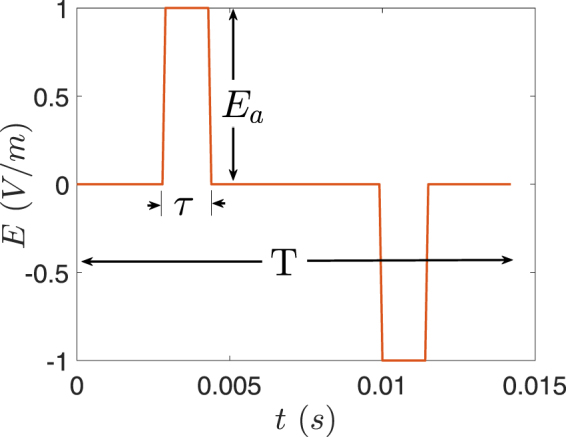
The biphasic waveform of the electric field. *E*_*a*_ is the amplitude; *τ* is the pulse width; and *T* is the period. The plot shows the specific case of frequency (1/*T*) 70 Hz and amplitude *E*_*a*_ = 1 V/m.

Since the capacitive effects were neglected when computing the electric potential in (1), the waveform of the electric field is similar to that of the electrode current. Thus, we considered the biphasic waveform shown in Fig. 10 by varying its parameters to cover the range of waveforms generated by ECT devices. Accordingly, the pulse width *τ* was varied from 0.25 ms–2 ms; the pulse frequency (*f* = 1/*T*) from 10 Hz–140 Hz; and the amplitude *E*_*a*_ was set to the maximum electric field in the foetal brain. The maximum electric field inside the foetal brain was compared to the ICNIRP basic restriction by converting it to the frequency domain via Fourier Transform and applying the spectral method outlined in the ICNIRP guideline^5^. For the electric field in the foetal brain to be below the ICNIRP basic restriction, the following equation should be satisfied

5 $$M(t)=|\sum _{i}\frac{{E}_{i}}{E{L}_{i}}\,\cos \,\mathrm{(2}\pi {f}_{i}t+{\theta }_{i}+{\phi }_{i})|\le \mathrm{1,}$$

where *t* is time and *EL*_*i*_ is the exposure limit at the ith harmonic frequency *f*_*i*_, where *E*_*i*_, *θ*_*i*_, *φ*_*i*_, are the amplitude of the electric field, its phase angles and phase angles of the filter (given in^5^) at the harmonic frequencies.

**Figure 11:**
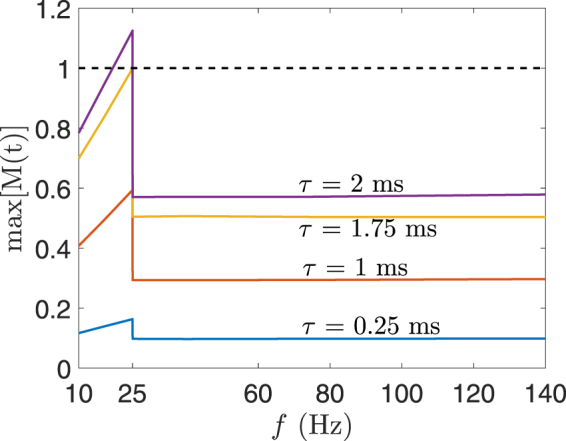
The maximum value of *M*(*t*) for the BL electrode placement when electrode current *I* = 0.9 A. The maximum value of *M*(*t*) was calculated for a range of electric field waveforms by varying the pulse width *τ* and pulse frequency *f*. The broken line indicates the ICNIRP basic restriction.

Figure 11 shows the maximum value of *M*(*t*) calculated for the electric field in the foetal brain for BL electrode placement when the electrode current is 0.9 A. Different waveforms were generated that have pulse widths in the range of 0.25 ms–2 ms and pulse frequency between 10 Hz–140 Hz. The electric field with waveforms of *τ* ≤ 1.75 ms was below the ICNIRP basic restriction for the whole pulse frequency range. Similarly, for BF, RUL, and LART electrode placement, the electric field obtained from all pulse widths and pulse frequencies is below the ICNIRP basic restriction. Moreover, the maximum electrode current that produces an electric field in the foetal brain below the ICNIRP basic restriction is 0.8 A for the range of pulse width and frequency considered. Given the specifications of ECT devices, the electric field produced inside the foetal brain is most likely below the ICNIRP basic restriction. For example, the MECTA devices (http://www.mectacorp.com) have a maximum current output of 0.8 A and pulse width of 0.3 ms–2 ms for the frequency range of 20 Hz–120 Hz. And the Somatics devices (http://www.thymatron.com) have a maximum current output of 0.9 A and pulse width 0.25 ms–1.5 ms for the frequency range of 10 Hz –140 Hz.

The electric field computed in this paper is for the specific case of a 30 weeks-old foetus that is in the bottom-up and head-down position and using the a computational model representing a pregnant female of height 1.62 m. It should be noted that the results do not represent the general case of different developmental stages of the foetus, different foetal positions, and the anatomical variations of the mother. Therefore, the readers should be cautious when using the results. The results serve to initiate further studies to establish more accurate and comprehensive assessments. The accuracy of the results can be improved by using a more anatomically realistic phantom of the pregnant female at different stages of pregnancy and more accurate conductivity values of tissues.

## Remarks

It might be worth mentioning the study conducted by Cech *et al*.^26^. The study compares the ICNIRP basic restriction and the electric current density produced inside the foetal brain when a uniform electric field (5 kV/m) and magnetic field (100 *μ*T) irradiate the whole-body of a 30-weeks pregnant voxel computational model. The electric and magnetic fields considered are the ICNIRP reference levels at 50 Hz. For the specific scenario considered, the study concluded that the current density produced inside the foetal brain exceeds the ICNIRP basic restriction. Our study focuses on a different scenario where the stimulus is an ECT pulse applied on the scalp (not a uniform electric or magnetic field irradiating the whole body); thus, naturally, the resulting conclusions are different.

## Conclusion

The electric field inside a 30 weeks-old foetus was computed using the computational model of a pregnant female. The dynamic impedance during ECT was represented by defining a current-dependent conductivity of the skin underlying the electrodes. Four types of electrode placement were simulated for electrode current of 0.9 A. Rectangular biphasic waveforms were considered with a range of pulse width (0.25 ms–2 ms) and frequency (10 Hz–140 Hz) that are commonly used in ECT devices. A linear relationship between the electric field inside the foetal brain and the electrode current was derived. The computed maximum electric field inside the foetal brain was compared to the basic restriction of ICNIRP. The results suggest that the electric field in the foetal brain from constant-current ECT devices is most likely below the ICNIRP basic restriction.
